# Development and evaluation of controlled porosity osmotic pump for Nifedipine and Metoprolol combination

**DOI:** 10.1186/1476-511X-10-51

**Published:** 2011-04-11

**Authors:** Rajagopal Kumaravelrajan, Nallaperumal Narayanan, Venkatesan Suba

**Affiliations:** 1 Department of Pharmaceutics, C.L. Baid Metha College of Pharmacy, Thoraipakkam, Chennai, Tamilnadu, India; 2 Department of Pharmaceutics, College of Pharmacy, Madras Medical College, Chennai, Tamilnadu, India; 3 Department of Pharmacology, National Institute of Siddha, Chennai, Tamilnadu, India

**Keywords:** Controlled porosity osmotic pump tablet, CPOP, Controlled osmotic drug delivery, Nifedipine, Metoprolol, Osmotic pump

## Abstract

**Background:**

A system that can deliver multi-drug at a prolonged rate is very important for the treatment of various chronic diseases such as diabetes, asthma and heart disease. Controlled porosity osmotic pump tablet (CPOP) system was designed to deliver Nifedipine (NP) and Metoprolol (MP) in a controlled manner up to 12 h. It was prepared by incorporating drugs in the core and coated with various types (PVP, PEG-400 and HPMC) and levels (30, 40 and 50% w/w of polymer) of pore former at a weight gain of 8, 12 & 15%.

**Results:**

Formulation variables like type and level of pore former and percent weight gain of membrane was found to affect the drug release from the developed formulations. Drug release was inversely proportional to the membrane weight but directly related to the level of pore former. Burst strength of the exhausted shell was inversely proportional to the level of pore former, but directly affected by the membrane weight. Results of scanning electron microscopy (SEM) studies showed the formation of pores in the membrane from where the drug release occurred. Dissolution models were applied to drug release data in order to establish the mechanism of drug release kinetics. *In vitro *release kinetics was subjected to superposition method to predict *in vivo *performance of the developed formulation.

**Conclusion:**

The developed osmotic system is effective in the multi-drug therapy of hypertension by delivering both drugs in a controlled manner.

## Background

Approximately one quarter of the total global population is affected by at least any one of the cardiovascular disease (CVD) [1]. It caused 2.3 million deaths in India in 1990, which may double by year 2020, where hypertension alone contribute 57% of all stroke death and 24% of all coronary heart disease [2]. Hypertension is a common cause of cardiovascular disorders and is essentially associated with abnormal lipid and altered glucose metabolism [3,4]. Several studies revealed that a reduction in blood pressure reduces the risk and incidence of CVD [5,6]. Thus management of cardiovascular disease in particular the hypertension becomes important to improve health care system. Several multidrug therapies are prescribed for successful management of CVD, in which chronic diseases such as hypertension, diabetes, asthma etc., are treated using multidrug therapies, which are vulnerable to incidences of side-effects, poor patient compliance and slow improvement of patients. Nifedipine (NP) and Metoprolol tartarate (MP) are anti-hypertensive agents belonging to calcium channel blockers and β-blockers respectively. Generally, they are either used individually or as combination therapy to treat hypertension. Though NP and MP is administered as immediate release solid oral dosage form, a short elimination half life with significant fluctuation in plasma concentration necessitate it to be formulated into modified release dosage forms. Recently, it was reported that smooth plasma profile of NP by modified release dosage forms decreased morbidity and mortality, prevents myocardial infarction in diabetes mellitus patients and reduces atherosclerosis in carotid and coronary arteries [7]. These facts justify our interest in controlled release dosage form. We have recently developed controlled release formulation of NP and MP based on sandwiched osmotic delivery system [8]. Osmotic drug delivery was attempted since, though a number of design options are available to control the drug release from a dosage form, majority of the oral dosage form fall in the category of matrix, reservoir or osmotic systems. Drug release from osmotic system is independent of pH and gastrointestinal motility to a large extent [9]. The development of oral osmotic systems has a strong market potential, as evident from the marketed products and number of patents granted in the last few years [10].

From a technical standpoint, the controlled delivery of NP is difficult since it is practically insoluble in water and aqueous fluids due to its high crystalline nature and exhibits poor dissolution rate. Various attempts were made to improve the dissolution rate of NP which include solid dispersion in water soluble carriers such as urea [11], PVP (Polyvinyl pyrrolidone) [12], PVP-MCC and HIPC-MCC [13] complexation with cyclodextrin. Skalko et al., [14] reported the feasibility of using NP-cyclodextrin complex as a core for microcapsule in order to obtain controlled release. Besides, recently Ramesh and Chowdary, [15] reported sustained release two layered tablet formulations of NP using NP-hydroxyproyl-β-cyclodextrin (1:1) inclusion complex. In the present investigation, 1:1 NP-hydroxyproyl-β-cyclodextrin (NHβ-CD) complex was used to improve NP dissolution rate and solubility.

There was a report of simultaneous delivery of drugs having extremely different solubility profile through elementary osmotic pump using highly water soluble drug as both osmotic agent and active ingredient to deliver it along with water insoluble drug [16]. Based on this report attempt was made to simultaneously deliver NP (water insoluble drug) and MP (highly water soluble drug) using the same approach. Instead, we tried controlled porosity osmotic pump (CPOP) system since preparation of CPOP is simple; it is not necessary to consider complicated side drilling and compared to other osmotic pump systems less excipients is required. The coating composition of CPOP includes pore-forming agent, which generates pores in contact with aqueous media [17]. It was observed that most of the core content releases through pores at a constant rate, where the release mechanism primarily is osmotic with simple diffusion playing a minor role. A zero-order delivery pattern was designed to produce plasma levels within the desired range. Different formulation variables were studied and optimized to achieve the desired release profile. Besides, the *in vivo *performance of the optimized formulation was predicted.

## Materials and methods

### Materials

Nifedipine (micronized) and Metoprolol tartarate were a kind gift sample from Madras Pharmaceuticals Private Limited, Chennai, India. Hydroxy propyl β-cyclodextrin (Hβ-CD) was purchased from Sigma-Aldrich, Bangalore. PVP (K-30) was purchased from s.d. Fine Chemicals Ltd, India. Hydroxy propyl methylcellulose (HPMC), Polyethylene glycol-400 (PEG-400) was obtained from Loba Chemie, Mumbai, India. Micro crystalline cellulose (MCC), magnesium stearate and aerosil were purchased from Rolex, Mumbai, India. Cellulose acetate (CA) was obtained from Eastman Chemical Company, Kingsport, U.S.A. All other solvents and chemicals used were of the analytical grade.

### Methods

#### Preparation of Nifedipine β-cyclodextrin inclusion complex

Solid complex of NHβ-CD were prepared in 1:1 ratio by co-precipitation method. NP was first dissolved in a small volume of acetone and then thoroughly mixed with 100 ml of ethanolic solution of carrier in a round bottom flask. The solvent was evaporated under reduced pressure at 40°C (Rotary evaporator RE120, Buchi, Switzerland) and stored in dessicator.

#### Preparation of tablets

Core tablets were prepared by wet granulation and the composition is given in table 1. For preparation of core tablets, the batch size was kept as 100 tablets. NHβ-CD and MP was mixed with starch and dicalcium phosphate and the blend was further mixed for 10 min. The mixture was moistened with PVP solution in isopropyl alcohol and granulated by passing through 12 sieve. The granules were dried at 50°C (approximately 1 h) after which they were passed through 18 sieve. These sized granules were then blended with magnesium stearate, talc and aerosil (all 120 sieve passed) and compressed into tablets using a rotary tablet compression machine (Cadmac, India) fitted with 12/32 inch deep concave punches. The core tablets were coated in an automated perforated pan (Cipweka, India). The composition of coating solution used for coating tablets is given in table 2. Various components of the coating solution were added to the solvent mixture in a sequential manner. The component added first was allowed to dissolve before the next component was added. Initially, pan was rotated at low speed (2-5 rev/min) and heated air was passed through the tablet bed. Coating process was started once the outlet air temperature reached 28°C. Coating pan rpm was kept in the range of 12-15 and coating solution was sprayed at the rate of 3-5 ml/min. Coating was continued until desired weight gain was obtained on the active tablets. In all the cases, active tablets were dried at 50°C for 2 h before further evaluation.

**Table 1:** Composition of core tablets of CPOP

Ingredients	Amount (mg)
NHβ-CD Complex	75
Metoprolol tartarate	55
Starch	10
Dicalcium phosphate	15
PVP	15
Isopropyl alcohol	Q.S
Aerosil	10
Talc	10
Magnesium stearate	02

**Table 2:** Formulation variables of controlled porosity osmotic pump tablet

Coating components	Formulation code							
	
	CF1	CF2	CF3	CF4	CF5	CF6	CF7	CFW
CA (% w/v)	4	4	4	4	4	4	4	4
PVP (% w/w of CA)	30	40	50	-	-	-		0
PEG 400 (% w/w of CA)	-	-	-	40		40	40	-
HPMC (% w/w of CA)	-	-	-	-	40	-	-	-
Thickness (%)	8	8	8	8	8	12	15	8

#### Evaluation of the developed formulations

The developed formulations were subjected to release studies using USP-II dissolution apparatus (Electrolab, India) at 100 rpm. Dissolution medium used was phosphate buffer (pH 6.8, 900 ml) containing sodium lauryl sulphate 0.5% maintained at 37 ± 0.5°C. The samples were withdrawn (10 ml) at 0, 2, 4, 6, 8, 10 and 12 h intervals and replaced with an equivalent amount of fresh medium. The dissolution sample after filtration through 0.45 μm cellulose acetate filter were analyzed using a validated UV spectrophotometric method at 340 nm for NP and 275 nm for MP. Each study was done in triplicate and the mean values were reported. After analyzing the drug content in the dissolution samples, the graph of cumulative percentage of drug release versus time was plotted. All experiments were carried out under strict protection from light to prevent undesirable photo-degradation of NP throughout the entire experimental procedure.

Mathematical comparisons of dissolution curves from various formulations provide an opportunity to test the similarity between two dissolution profiles. Fit factors (*f*_1 _and *f*_2_) proposed by Moore and Flanner, [18] were used for comparing the dissolution profile of pH and agitation studies. An *f*_2 _value of ≥50 indicates similarity between two dissolution curves, whereas *f*_1 _is used as an additional parameter to confirm the similarity when the value is ≤15. For the calculation of *f_1 _*and *f_2 _*values, only one data point was taken into consideration after 85% of the drug was released.

#### Formulation variables

In order to optimize the formulation to release the drug at a constant zero-order release rate, level of pore former, type of pore former and coating thickness were used as formulation variables [19]. The respective formulation code and variables were given in table 2

#### Effect of type and level of pore former

To study the effect of pore former on the drug release, PVP, PEG-400 and HPMC were used as pore former at the level of 0, 30, 40 and 50% (w/w) of CA.

#### Effect of coating thickness on drug release

The tablets coated to three level of weight gain, such as 8, 12 and 15% (w/w) in order to assess the effect of coating thickness on drug release.

#### Burst strength

Burst strength of the exhausted shells, after dissolution was determined to assure that the tablets would maintain their integrity in the gastrointestinal tract (GIT). Burst strength was determined as the force required to break/rupture the shells after dissolution studies. The texture analyzer (Instron 4501, India) with a 5 kg load cell and 25 mm aluminum cylindrical probe was utilized for this purpose. Test speed of 0.8 mm/s with a distance of 2 mm was selected.

#### Scanning electron microscopy studies

In order to elucidate the mechanism of drug release from the developed formulations, surface of coated tablets before and after dissolution studies, was studied using scanning electron microscope (SEM). The samples were placed on a spherical brass stub (12 mm diameter) with a double backed adhesive tape. Small sample of the coating membrane was carefully cut from the exhausted shells (after dissolution studies) and dried at 50°C for 2 h. The mounted samples were sputter coated for 2 min with gold using fine coat ion sputter (JFC-1600, Jeol, Japan) with pressure of 8 kg Pascal and examined under SEM (JSM-6360, Jeol, Japan).

#### Effect of pH

In order to study the effect of pH and to assure a reliable performance of the developed formulations independent of pH, release studies of the optimized formulations were conducted in media of different pH, simulated gastric fluid (SGF) pH 1.2, simulated intestinal fluid (SIF) pH 6.8 and pH change method (release media was SGF for first 2 h, followed by SIF for the remaining period). The sample (10 ml) was withdrawn at pre-determined intervals and analyzed after filtration through 0.45-μm cellulose acetate filter. The percentage cumulative drug release of optimized formulations at various pH was plotted and compared.

#### Effect of agitational intensity

In order to study the effect of agitational intensity of the release media, release studies of the optimized formulations were carried out in dissolution apparatus at various rotational speeds. Dissolution apparatus used was USP-II at 50, 100 and 150 rpm. Sample was withdrawn at pre-determined intervals and analyzed after filtration through 0.45 μm cellulose acetate filter. The percentage cumulative drug release of optimized formulations at different agitational intensity was plotted and compared.

#### Osmotic pressure measurement

In order to confirm the mechanism of drug release, release studies of the optimized formulations were conducted in media of different osmotic pressure. To increase the osmotic pressure of the release media, sodium chloride (Osmotically effective solute) was added in SIF [20] and osmotic pressure was measured (Fiske micro osmometer, 210). The pH was adjusted to 6.8 ± 0.05. Release studies were carried out in 900 ml of media using USP II dissolution test apparatus (100 rpm). Release profiles of the optimized formulations at different osmotic pressure was plotted and compared.

#### Curve fitting analysis

In order to describe the kinetics of drug release from controlled release preparations various mathematical equations have been proposed. Release data obtained was applied to different release models in order to establish the drug release mechanism and kinetics. Best goodness of fit test (R^2^) was taken as criteria for selecting the most appropriate model [21].

#### In vivo performance prediction of selected formulation

Drug-release parameters (R^0 ^and t_del_) obtained from *in vitro *data and the pharmacokinetic properties of drugs were used for predicting blood drug concentration-time profile from single dose and at steady state from multiple dosing. The method of superposition was used for the steady-state concentration predictions [22]. It was assumed that after the administration of a test dose of formulation, the drug would be released at a release rate (R^0^) for a period of time (t_del_) shorter than the selected dosing interval (*τ*). Values of C_ssmax _and C_ssmin_, maximum and minimum steady-state concentration, respectively, were compared with the desired values calculated from a theoretically developed controlled drug-release profile [23]. The predicted steady-state plasma level of developed formulation was compared with the desired levels by calculating the percent predicted error (% PD) in C_SSmax_, C_ssmin _and AUC_0-τ_. Bioequivalence was anticipated [24] if the average % PD was less than 15%. The % PD was calculated using the following equation:

## Result and Discussion

### Desired drug release profile

The purpose of this study was to develop a delivery system for short half life drugs NP and MP which can deliver these drugs in a controlled manner for 12 h. The dose, the delivery time and the dosing interval are the key features for any temporal controlled release system. The desired steady-state concentration for NP to the therapeutically acceptable range is 15-75 ng/ml [25]. In the present study, steady state concentration was selected as 15-60 ng/ml. Similarly, MP steady state plasma concentration was reported as 25-46 ng/ml [26]. The same plasma concentration was selected in this study. Taking different pharmacokinetic parameters of NP and MP into consideration, a zero-order based delivery strategy was designed to produce the desired plasma levels [22]. Series of simulations (using Microsoft Excel 2003) were performed and it was found that a delivery rate of 1.68 mg/h for a period of 5.19 h and 4.2 mg/h for a period of 8.8 h was found to meet the above requirements for NP and MP respectively.

### Formulation development

**Figure 1:**
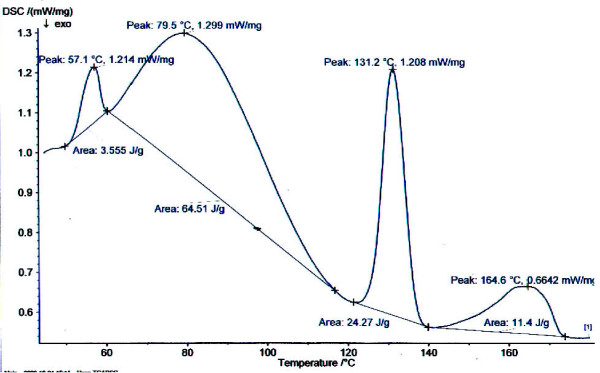
**DSC thermogram of NHβ-CD and meoprolol combination CPOP**.

The compatibility of NP, MP with the excipients used for formulation development was tested using differential scanning calorimetry (DSC). The changes in the endotherm observed in case of CPOP tablet (melting endotherm of NHβ-CD at 154.6°C and MP at 131.2^°^C, Figure 1) for the original melting endotherm (figure not shown) of NHβ-CD (169.7^°^C) and MP (146.27^°^C) was minor. Hence it was clear that no specific interaction between the drug and excipients used in the present formulation. In general, both highly and poorly water-soluble drugs are not good candidates for osmotic delivery. As previous study demonstrated increased solubility of NP by complexation with Hβ-CD, we also prepared inclusion complex of NHβ-CD (1:1) by co-precipitation method. Solubility of NP in distilled water at 25°C was 6.24 μg/ml whereas the solubility of prepared complex was found to be 15 μg/ml. Phase solubility studies of NHβ-CD systems in water at 25°C revealed that the solubility of NP increased linearly with the increase in the concentration of Hβ-CD, showing a typical A_L_-type phase solubility curve. This curve may be ascribed to the formation of a stoichiometric 1:1 complex of NP and Hβ-CD. The apparent 1:1 stability constant (K_C_) was calculated from the straight line of the phase solubility diagram and constant value was found to be 10.35 M^-1^.

The dosage form developed consists of a tablet core of NHβ-CD and MP along with other excipients. Tablet core consists of drugs and other conventional excipients to form the core compartment. The core compartment is surrounded by a membrane consisting of a semipermeable membrane-forming polymer, water-soluble pore-forming additives and at least one plasticizer capable of improving film forming properties of the polymers. The semipermeable membrane-forming polymer is permeable to aqueous fluids but substantially impermeable to the components of the core. In operation, the core compartment imbibes aqueous fluids from the surrounding environment across the membrane and dissolves the drug. The dissolved drugs are released through the pores created after leaching of water-soluble additive(s) in the membrane. CA was used as water insoluble polymer. PVP, PEG-400 and HPMC were tried as pore formers. Diethyl phthalate (DEP) 20% was used as a plasticizer. We attempted to deliver NP and MP by adding highly water soluble MP in to the NHβ-CD tablets as both active agent and also as an osmotic agent. The release of NP was enhanced owing to osmotic effect of MP and further MP was also delivered simultaneously.

### Influence of tablet formulation variables on NP and MP release

To study the influence of tablet formulation variables on drug release, tablets with various formulations were prepared, subsequently coated with the coating thickness of 8% and plasticized with 20% DEP.

### Effect of level of pore former

**Figure 2:**
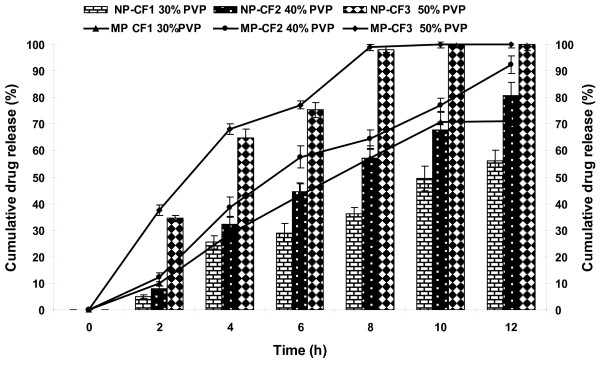
**Effect of level of pore former on release of NP and MP**.

To study the effect of level of pore former (PVP), core tablets were coated with coating composition containing 0, 30, 40 and 50% (w/w) of PVP (formulations: CFW, CF1, CF2 and CF3 respectively). It was found that the drug release increased with increase in the level of pore former (Figure 2). As the level of pore former increases, the membrane becomes more porous after coming in contact with the aqueous environment, resulting in faster drug release. Other workers have also obtained similar results [27,28]. At levels up to 30% and 40% (w/w) of pore former, numbers of pores were sufficient to contribute to significant drug release. On the other hand, membrane that contained 50% (w/w) of pore former; the release profile was faster since it became highly porous after coming in contact with water. Another parameter affected by the level of pore former was burst strength of the exhausted shells. The burst strength was inversely related to the initial level of pore former in the membrane. With the increase in level of PVP, the membrane became more porous after exposure to water, leading to decrease in its strength. Effect of level of PVP on burst strength is shown in table 3. Since, satisfactory drug release and adequate burst strength were obtained in case of formulations with 40% pore former level (CF2); this concentration was selected for further studies.

**Table 3:** Effect of level and type of pore former on burst strength

S.No	Pore former (% w/w of CA)	Burst strength (MPa)
1	PVP (0)	50
2	PVP (30)	14.25
3	PVP (40)	8.18
4	PVP (50)	5.31
5	PEG-400 (40)	18.25
6	HPMC (40)	8.37

### Effect of type of pore former

**Figure 3:**
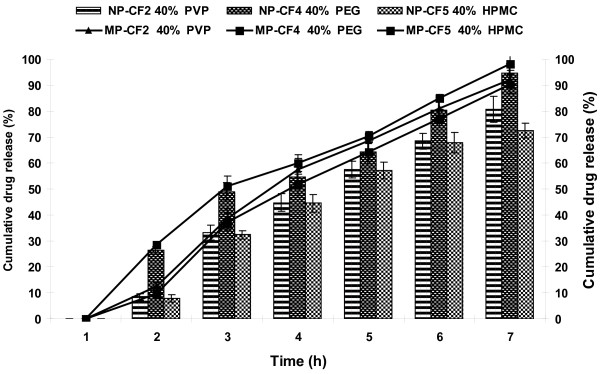
**Effect of type of pore former on release of NP and MP**.

To study the effect of type of pore former, formulations CF2, CF4 and CF5 were prepared by coating core tablets of NP and MP with coating compositions containing different pore formers PVP 40% (CF2), PEG-400 40% (CF4) and HPMC 40% (CF5). As evident from Figure 3, the type of pore former affected drug release and it is possible to achieve the desired release by using different types of pore formers. It has been reported that water-soluble polymers such as PVP, PEG and HPMC may leach out of the coating, forming a porous film with increased permeability [29] or produce hydrated water filled HPMC regions within the membrane that allow drug transport across the film. The percentage drug release was more in case of PEG-400 as pore former, since it is more hydrophilic plasticizer than PVP and HPMC, it could be leached out easily and increases the flux rate of fluid. The poor drug release pattern of HPMC may result from the increased tortuosity or gel strength of the polymer. In addition to release, type of pore former also affected the burst strength of the exhausted shells (table 3) and this parameter should also be taken into consideration while selecting the pore former. The drug release pattern and the burst strength were satisfactory with the formulations containing PEG-400 (CF4) as the pore former. This formulation was selected as the optimized formulation and used for further evaluation.

### Effect of weight gain

**Figure 4:**
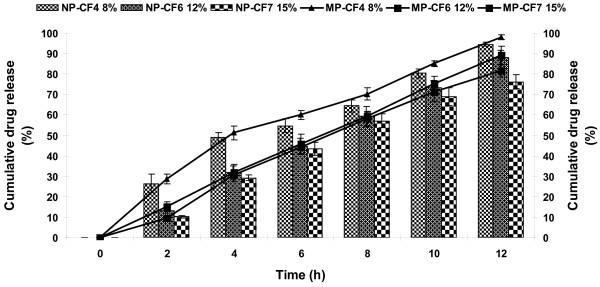
**Effect of weight gain on release of NP and MP**.

**Figure 5:**
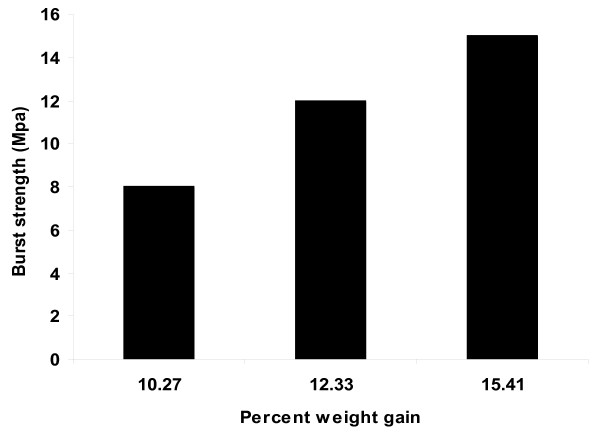
**Burst strength of the membrane as function of weight gain**.

To study the effect of weight gain of the membrane, coating on the core tablets of NP was continued for sufficient duration so as to get tablets with different weight gain 8 (CF4), 12 (CF6) and 15% (CF7). Release profile of NP and MP as a function of weight gain of the membrane is shown in Figure 4. Drug release was found to decrease with an increase in the weight gain of the membrane. The burst strength of these formulations were also observed (Figure 5). Though CF4 and CF6 showed higher drug release pattern, comparatively an approximate zero order release pattern and better burst strength was observed with CF6. In case of 15% (CF7) thickness slow release pattern was observed. This is due to the increased polymer loading. The more the weight gain, the more would be the lag time. No bursting of the systems was observed during the dissolution run in any of the formulations. In addition, exhausted tablets (after 12 h of dissolution studies) were evaluated for burst strength to assure that the tablets maintain their integrity in GIT and do not lead to dose dumping.

The strength of mechanical destructive forces in the GIT of humans and dogs has been reported to be 1.9 N (approximately 190 g) and 3.2 N (approximately 320 g), respectively [30,31]. In a previous study, it has been reported that osmotic pumps having the burst strength in the range of 500-600 g were intact in the GIT of dogs while those having burst strength of around 200 g were compromised [32]. In all cases of our investigation the value of burst strength is much higher than the mechanical destructive forces in GIT, thus assuring that the formulations can be expected to remain intact in GIT without any incidence of dose dumping. It was found that an approximate zero-order release rate pattern up to 12 h and sufficient burst strength was obtained in the case of the 12% coating thickness. Hence CF6, PVP-40% and weight gain of 12% was used as optimized formulation for further evaluations.

### Kinetics and Mechanism of drug release

Drug-release data from the optimized formulation was fitted to various kinetic models to elucidate the mechanism and kinetics of drug release (table 4). Best goodness of fit test (R^2^) was taken as criteria for selecting the most appropriate model. The data fit well into the zero order kinetics. The compatible fit of the zero order kinetics indicated that the drug release is controlled by a concentration independent release mechanism.

**Table 4:** Fitting of NP and MP release data of the optimized formulation (CF6) according to various mathematical models

Model	Parameters used to assess the fit of model							
	
	R^2^		Intercept		K		AIC	
	
	NP	MP	NP	MP	NP	MP	NP	MP
**Zero order**	0.9986	0.9994	0.239	0.741	1.48	3.94	5.08	-0.99
**First order**	-0.9174	-0.9134	2.09	2.09	-0.16	-0.17	-30.32	-29.39
**Higuchi**	0.9649	0.9671	-11.68	-11.35	25.6	25.84	33.92	33.72

R^2 ^-Goodness of fit.

K - Release rate constant for respective models (K_0 _in mg/hr, K_1 _in h^-1 ^and K_H _in % h^1/2 ^for zero order, first order and Higuchi rate equations respectively).

AIC - Akaike information criterion

**Figure 6:**
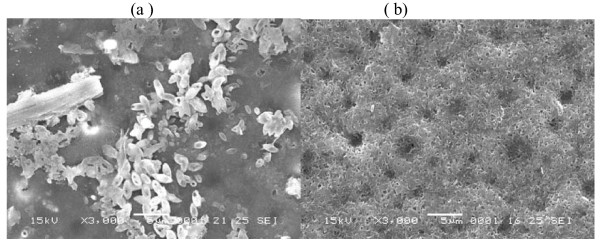
**SEM micrograph of 40% PVP (a) before and (b) after dissolution studies**.

To investigate the changes in the membrane structure, surface of coated tablets was studied using SEM. Figure 6, 7 &8 showed SEM micrographs of membrane surface of developed formulations CF2, CF4 and CF5 containing 40% of PVP, PEG-400 and HPMC before and after dissolution studies. After dissolution studies, coating was intact without any cracks. However, there was formation of channels/pores in the membrane, which possibly acted as exit ports for the drug.

**Figure 7:**
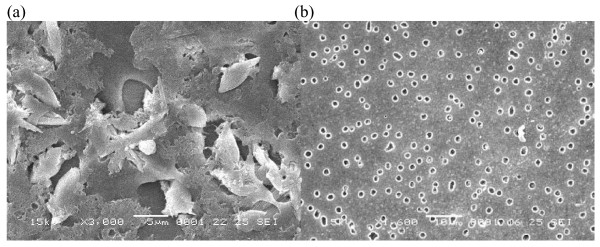
**SEM micrograph of 40% PEG-400 before (a) and after (b) dissolution studies**.

**Figure 8:**
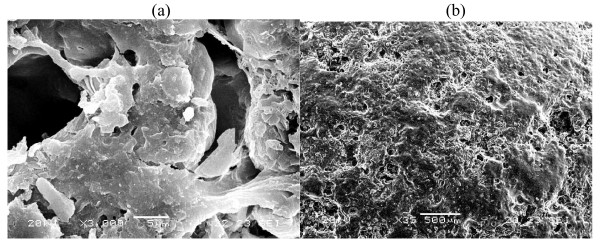
**SEM micrograph of 40% HPMC before (a) and after (b) dissolution studies**.

Figure 6a, 7a and 8a showed the membrane structure before dissolution studies. In all the cases, no significant difference was observed between different types of pore former (PVP, PEG-400 and HPMC). The surface of coated tablet was smooth before coming into contact with the aqueous environment and the coats appeared to be free of point defects.

When comparison was made among the membrane containing different types of pore formers after dissolution studies, it was found that the membrane that contained PVP (Figure 6b), PEG-400 (Figure 7b) and HPMC **(**Figure 8b) developed pores. But the size and number of pores are more in case of PEG-400. The membrane becomes more porous, because of leaching of pore former from the membrane.

### Effect of pH

In order to study the effect of pH on drug release, release studies were conducted in media of different pH. To study the effect of pH and to assure a reliable *in-vivo *performance, release study of the optimized formulation was conducted according to pH change method and compared with release profile in SIF (pH 6.8). Table 5 showed release of NP and MP from optimized formulation of CPOP (CF6) in pH 1.2; pH change method and pH 6.8 respectively. The *f*_1 _and *f*_2 _values were found to be 3 and 78 (between pH 1.2 and pH 6.8), 3 and 76 (between pH change method and pH 6.8) for NP and 2 and 81 (between pH 1.2 and pH 6.8), 3 and 77 (between pH change method and pH 6.8) for MP respectively. As can be seen from table 5, release profile is similar in all the media demonstrating that the developed formulations show pH-independent release.

**Table 5:** Effect of pH on release of NP and MP from CF6 (n = 3)

Time (h)	Cumulative % release ± SEM					
	
	NP			MP		
	
	pH 6.8	pH 1.2	pH change	pH 6.8	pH 1.2	pH change
0	0.00 ± 0.00	0.00 ± 0.00	0.00 ± 0.00	0.00 ± 0.00	0.00 ± 0.00	0.00 ± 0.00
2	13.28 ± 0.68	12.60 ± 1.30	13.40 ± 0.71	15.31 ± 1.71	12.52 ± 2.31	12.02 ± 1.35
4	31.81 ± 1.23	29.50 ± 1.89	27.40 ± 1.12	31.71 ± 1.20	28.50 ± 2.44	26.50 ± 2.50
6	44.74 ± 1.50	41.31 ± 2.80	39.08 ± 1.54	45.66 ± 3.12	42.12 ± 2.09	40.82 ± 1.51
8	59.28 ± 1.91	55.90 ± 2.30	53.62 ± 2.31	59.41 ± 2.96	56.95 ± 2.61	53.40 ± 2.97
10	73.15 ± 0.21	70.12 ± 2.67	67.93 ± 3.02	75.27 ± 2.31	72.85 ± 3.42	70.16 ± 2.46
12	88.14 ± 1.10	85.41 ± 1.80	85.20 ± 2.86	89.13 ± 1.90	87.09 ± 3.79	86.40 ± 1.83

### Effect of agitational intensity

Drug release from osmotic pumps to a large extent is independent of agitation intensity of the release media. To study the effect of agitational intensity of the release media, release studies of the optimized formulation of CPOP (CF6) were carried out in USP dissolution apparatus type II at varying rotational speed (50, 100 and 150 rpm). It is clearly evident from table 6 that the release of NP and MP from CPOP is independent of the agitational intensity. The *f*_1 _and *f*_2 _values were found to be 5 and 69 (between pH 1.2 and pH 6.8), 4 and 69 (between pH change method and pH 6.8) for NP and 4 and 67 (between pH 1.2 and pH 6.8), 3 and 77 (between pH change method and pH 6.8) for MP respectively. Hence, it can be expected that the release from the developed formulation will be independent of the hydrodynamic conditions of the absorption site.

**Table 6:** Effect of agitational intensity on release of NP and MP from CF6 (n = 3)

	Cumulative % of release ± SEM					
	
	NP			MP		
	
Time (h)	100 RPM	50 RPM	150 RPM	100 RPM	50 RPM	150 RPM
0	0.00 ± 0.00	0.00 ± 0.00	0.00 ± 0.00	0.00 ± 0.00	0.00 ± 0.00	0.00 ± 0.00
2	8.50 ± 0.71	13.28 ± 0.68	14.50 ± 1.30	11.41 ± 2.31	15.31 ± 1.71	18.21 ± 1.35
4	27.35 ± 1.12	31.81 ± 1.23	34.21 ± 1.89	27.80 ± 2.44	31.71 ± 1.20	33.4 ± 2.5
6	38.46 ± 1.54	44.74 ± 1.50	47.41 ± 2.80	41.04 ± 2.09	45.66 ± 3.12	49.50 ± 1.51
8	55.92 ± 2.31	59.28 ± 1.91	62.53 ± 2.30	55.71 ± 2.61	59.41 ± 2.96	63.21 ± 2.97
10	65.13 ± 3.02	73.15 ± 0.21	77.08 ± 2.67	71.58 ± 3.42	75.27 ± 2.31	77.81 ± 2.46
12	83.53 ± 2.86	88.14 ± 1.10	91.73 ± 1.80	85.02 ± 3.79	89.13 ± 1.90	93.41 ± 1.83

### Effect of osmotic pressure

The effect of osmotic pressure on the optimized formulation was studied in media of different osmotic pressure, and the release profile with varying osmotic pressure is depicted in table 7. The results of release studies of optimized formulations in media of different osmotic pressure indicated that, the drug release is highly dependent on the osmotic pressure of the release media. NP and MP release from the formulations decreased as the osmotic pressure of the media increased. The release was inversely related to the osmotic pressure of the release media. This finding confirms that the mechanism of drug release is by the osmotic pressure.

**Table 7:** Effect of osmotic pressure on release of NP and MP from CF6

Time (h)	Cumulative % of drug release							
	
	NP				MP			
	
	CF6	7.8 atm	13.85 atm	21.27 atm	CF6	7.8 atm	13.85 atm	21.27 atm
0	0.00	0.00	0.00	0.00	0.00	0.00	0.00	0.00
2	13.28	8.50	5.20	3.10	15.31	10.20	6.21	3.45
4	31.81	24.30	18.40	12.30	31.71	24.60	18.20	12.00
6	44.74	33.20	30.10	22.40	45.66	38.60	29.50	21.40
8	59.28	48.40	41.50	29.50	59.41	50.00	39.50	30.20
10	73.15	65.00	56.40	45.30	75.27	66.14	58.70	50.36
12	88.14	79.10	68.00	58.60	89.13	79.50	70.13	64.20

### Prediction of in vivo concentration-time profile from in vitro data

**Figure 9:**
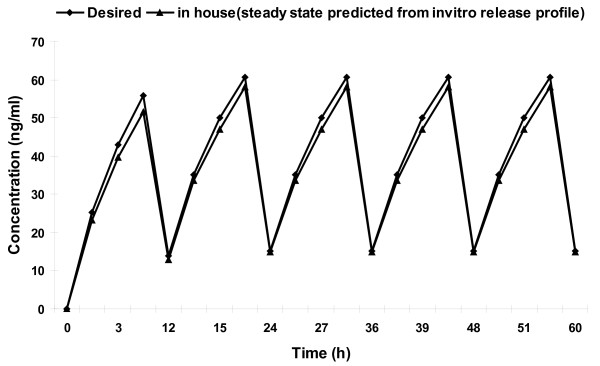
**Predicted steady-state concentration of NP in comparison with the desired profile**.

**Figure 10:**
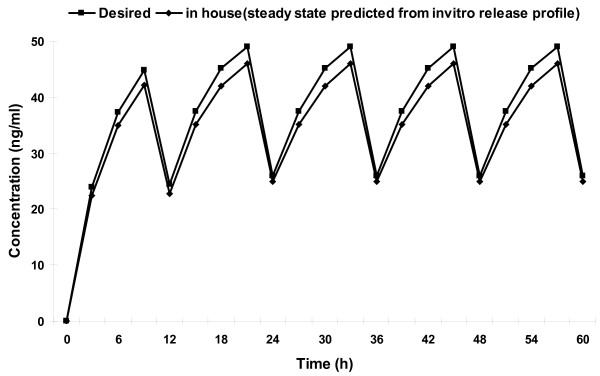
**Predicted steady-state concentration of MP in comparison with the desired profile**.

Method of superposition was used to predict steady state plasma levels of NP and MP after administration of CF6 formulation [22]. A steady state drug concentration-time profile for the optimized tablet was predicted using its *in vitro *zero order kinetic release rate. Because the release rate of drugs from the tablets was slightly lower than the desired level, the predicted drug concentrations were also proportionately lower to the desired concentrations. Prediction of steady-state levels of NP and MP after administration of a test dose of formulation showed that peak plasma levels were 58 (NP) and 45.98 ng/ml (MP) but falls to14.8 (NP) and 25 ng/ml (MP) at steady state (Figure 9, 10). The predicted C_SSmax_, C_SSmin _and AUC_0- __τ _after administration of formulation of NP and MP, in comparison with the desired ones are listed in table 8. The % PD of the steady-state parameters of CF6 formulation was calculated taking the data of desired profile as the reference. The absolute % PD was found to be less than 15%, ensuring that the formulation will produce plasma levels close to the desired ones. Thus, it can be concluded that the developed formulation (CF6) will produce plasma levels well within the therapeutic range and similar to those produced by the desired zero order delivery profile.

**Table 8:** Predicted *in vivo *performance of the developed formulation for NP and MP

Predicted parameters	NP		PD (%)	MP		PD (%)
	Desired^a^	Formulation^b^*		Desired^c^	Formulation^d^*	
**Cmax (ng/ml) after a single dose study**	55.85	51.83	-7.2	44.85	42.08	-6.1
**Cmax (ng/ml) at steady state**	60.68	58.0	-4.4	49	45.98	-6.1
**Tmax (h)**	5.19	5.18	-.19	8.921	8.906	-0.16
**Cmin (ng/ml) at steady state**	15.16	14.8	-2.3	26	25	-3.8
**AUC_0-t _(ng h/ml) after a single dose study**	405	374	-7.6	357	334	-6.4
**AUC_0-t _(ng h/ml) at steady state**	669	639	-4.6	661	619	-6.3

**^a ^**Predicted from desired zero-order delivery profile (NP) (dose = 20 mg, *R*^0 ^= 1.677 mg/h, and *t*_del _= 5.19 h).

^b^*Predicted from drug release studies (NP-Formulation CF6) (dose = 20.13 mg, *R*^0 ^= 1.479 mg/h, and *t*_Del _= 5.18 h).

**^c^**Predicted from desired zero-order delivery profile (MP) (dose = 50 mg, *R*^0 ^= 4.20 mg/h, and *t*_Del _= 8.79 h).

^d^*Predicted from drug release studies (MP-Formulation CF6) (dose = 50.49 mg, *R*^0 ^= 3.94 mg/h, and *t*_Del _= 8.90 h).

## Conclusion

In the present study an oral osmotic system was developed that can deliver NP and MP simultaneously. This study suggests that drug release from these systems is controlled by osmotic pressure as the major mechanism; release pattern followed zero order kinetics and independent of environmental medium and the mobility of the gastrointestinal tract. The feasibility of extending the zero order release pattern of both the drugs were better achieved with controlled porosity osmotic pump tablet system between 10-12 h. The prototype design of the system could be applied to other combinations of drugs (one slightly water soluble or insoluble drug and another freely water soluble drug) used in cardiovascular diseases, diabetes etc.

## Conflict of interests

The authors declare that they have no competing interests.

## Authors' contributions

RK carried out the formulation, *in vitro *characterization and drafted the manuscript. NR participated in its design and coordination and helped to draft the manuscript. VS helped out in the prediction of *in vivo *performance from the *in vitro *release characteristic studies. All authors read and approved the final manuscript.
